# Supplementary material for: Synthesis and Spectroscopic Characterization of Selected Water-Soluble Ligands Based on 1,10-Phenanthroline Core
Source: Molecules. 2024 Mar 18;29(6):1341. doi: 10.3390/molecules29061341 (PMC10975036; doi:10.3390/molecules29061341)
Supplement: Supplementary file 1 [file molecules-29-01341-s001.zip › Supporting Information Nycz.pdf]

Supporting Information for

Synthesis and Spectroscopic Characterization of Selected Water-Soluble Ligands Based on 1,10-phenanthroline Core

Jacek E. Nycz [1,*], Natalia Martsinovich [2], Jakub Wantulok [1], Tieqiao Chen [3], Maria Książek [4] and Joachim Kusz [4]

[1] Institute of Chemistry, Faculty of Science and Technology, University of Silesia in Katowice, ul. Szkolna 9; 40-007 Katowice, Poland; jakub.wantulok@us.edu.pl (J.W.)
[2] Department of Chemistry, University of Sheffield, Sheffield, UK; n.martsinovich@sheffield.ac.uk (N.M.)
[3] Key Laboratory of Ministry of Education for Advanced Materials in Tropical Island Resources, Hainan Provincial Key Lab of Fine Chem, Hainan Provincial Fine Chemical Engineering Research Center, Hainan University, Haikou, 570228, China; chentieqiao@hnu.edu.cn (T.C.)
[4] Institute of Physics, Faculty of Science and Technology, University of Silesia in Katowice, 75 Pułku Piechoty 1a, 41-500 Chorzów, Poland; maria.ksiazek@us.edu.pl (M.K.), joachim.kusz@us.edu.pl (J.K.)

Table of Contents

Figures S1–S2 computational analysis S2-S3
Figures S3-S11. ^{1}H, $^{13}C\{^{1}H\}$ NMR, MS and HRMS spectra of the compounds S4-S25

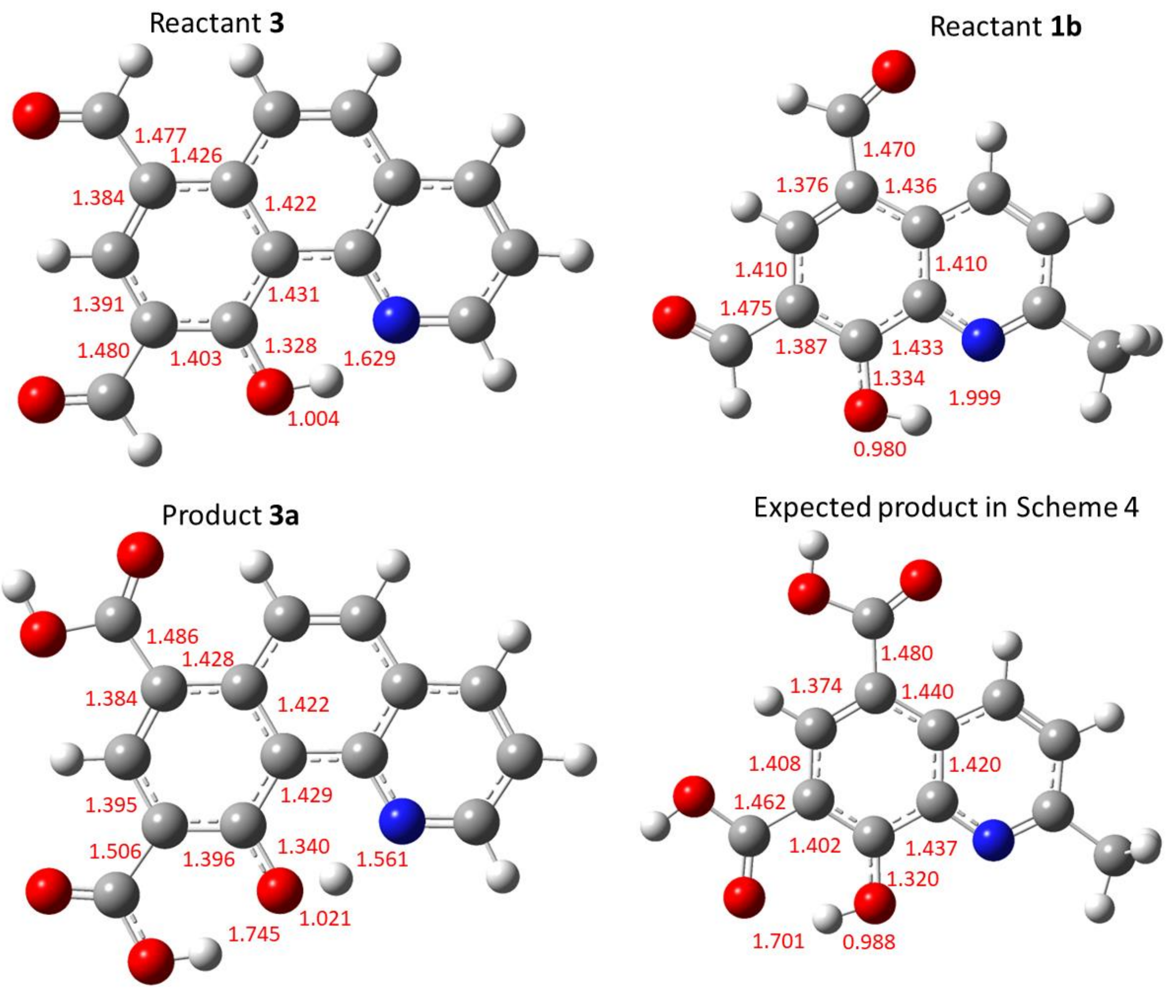

Figure S1: Calculated structures and bond distances of compounds **3**, **3a** (Scheme 1) **1b** (Scheme 3) and the expected diacid product in Scheme 3.

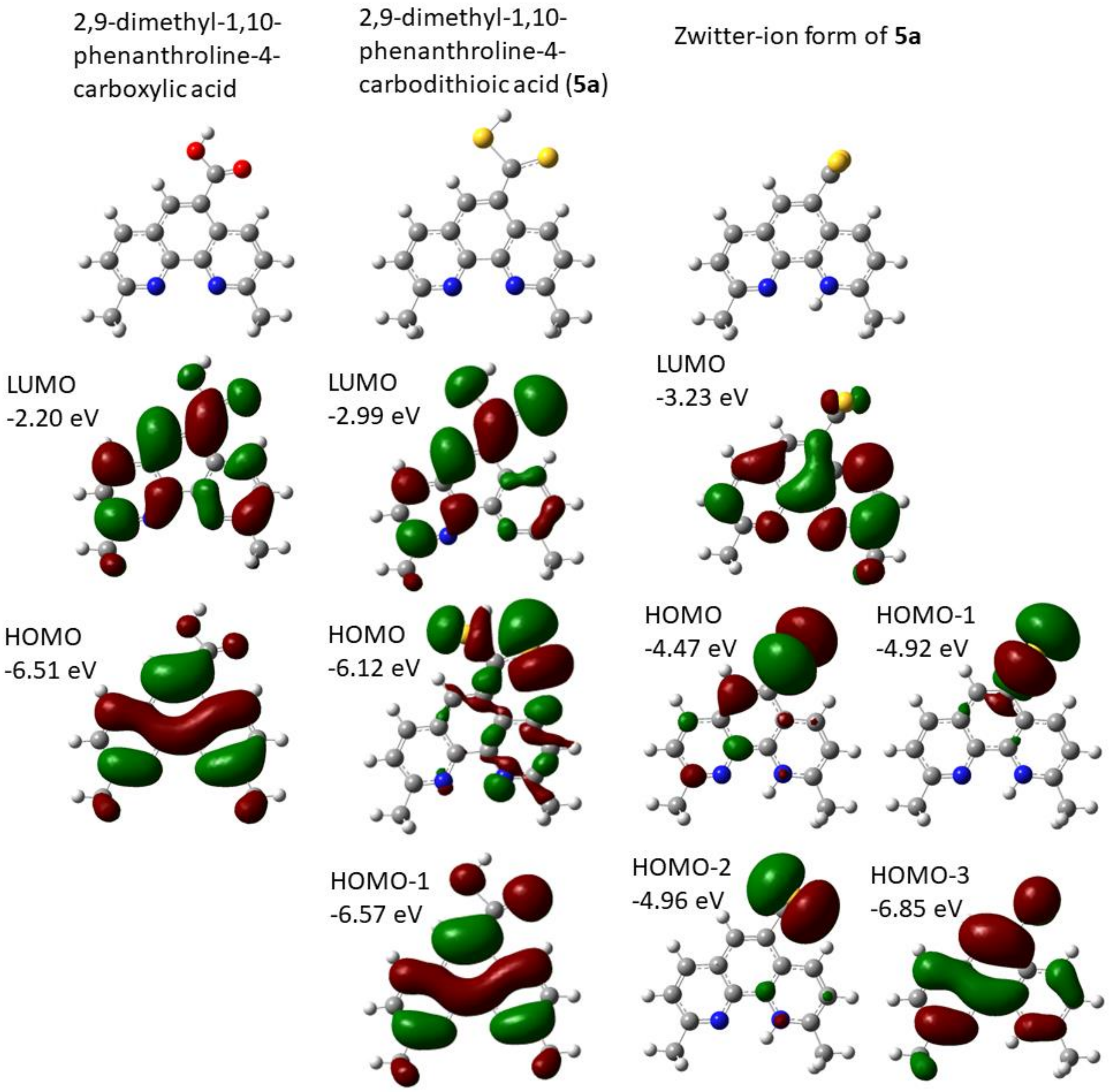

Figure S2: Structures and frontier molecular orbitals of 2,9-dimethyl-1,10-phenanthroline-4-carboxylic acid in its neutral form, and 2,9-dimethyl-1,10-phenanthroline-4-carbodithioic acid (**5a**) in its neutral and zwitter-ion form.

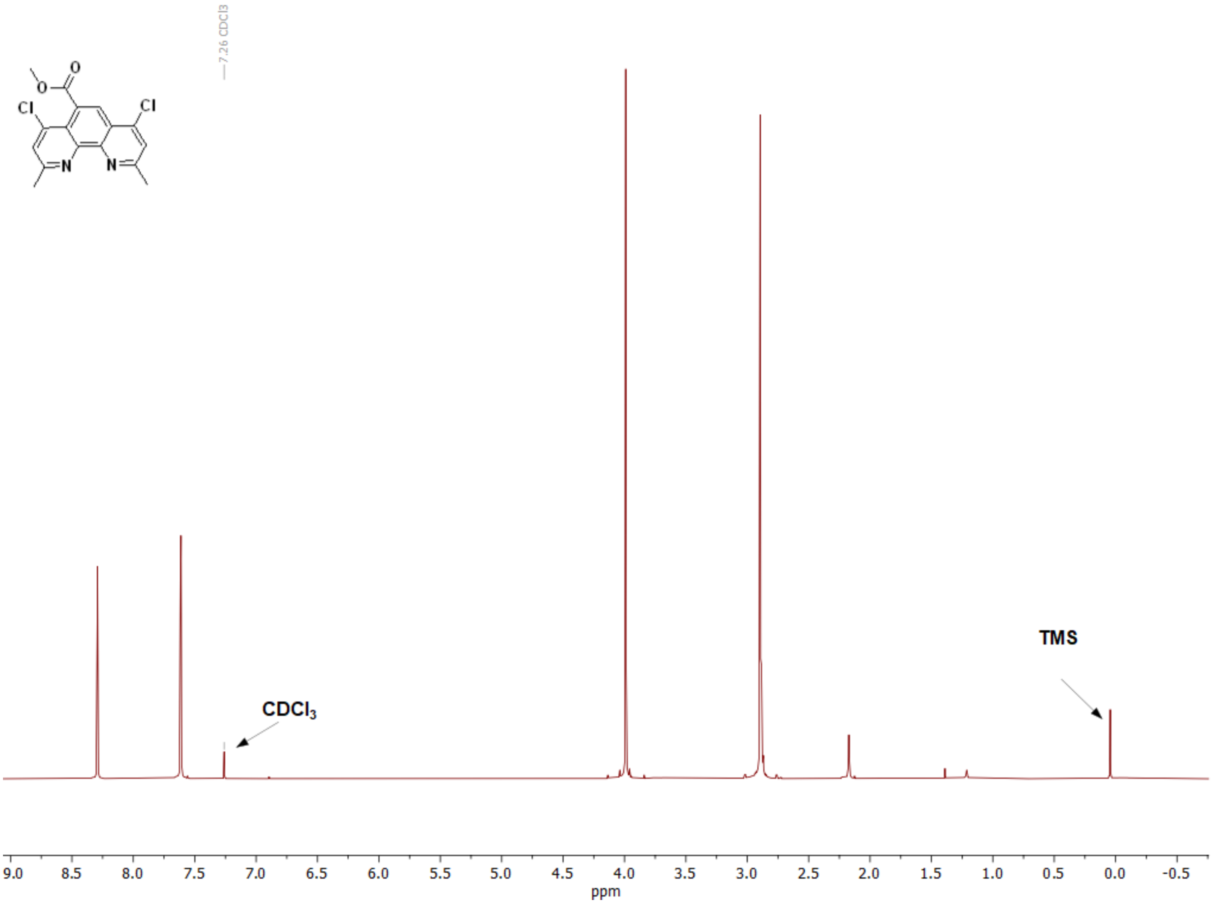

Fig. S3a. ^{1}H-NMR ($CDCl_3$; 500.2 MHz) spectrum of **1d**.

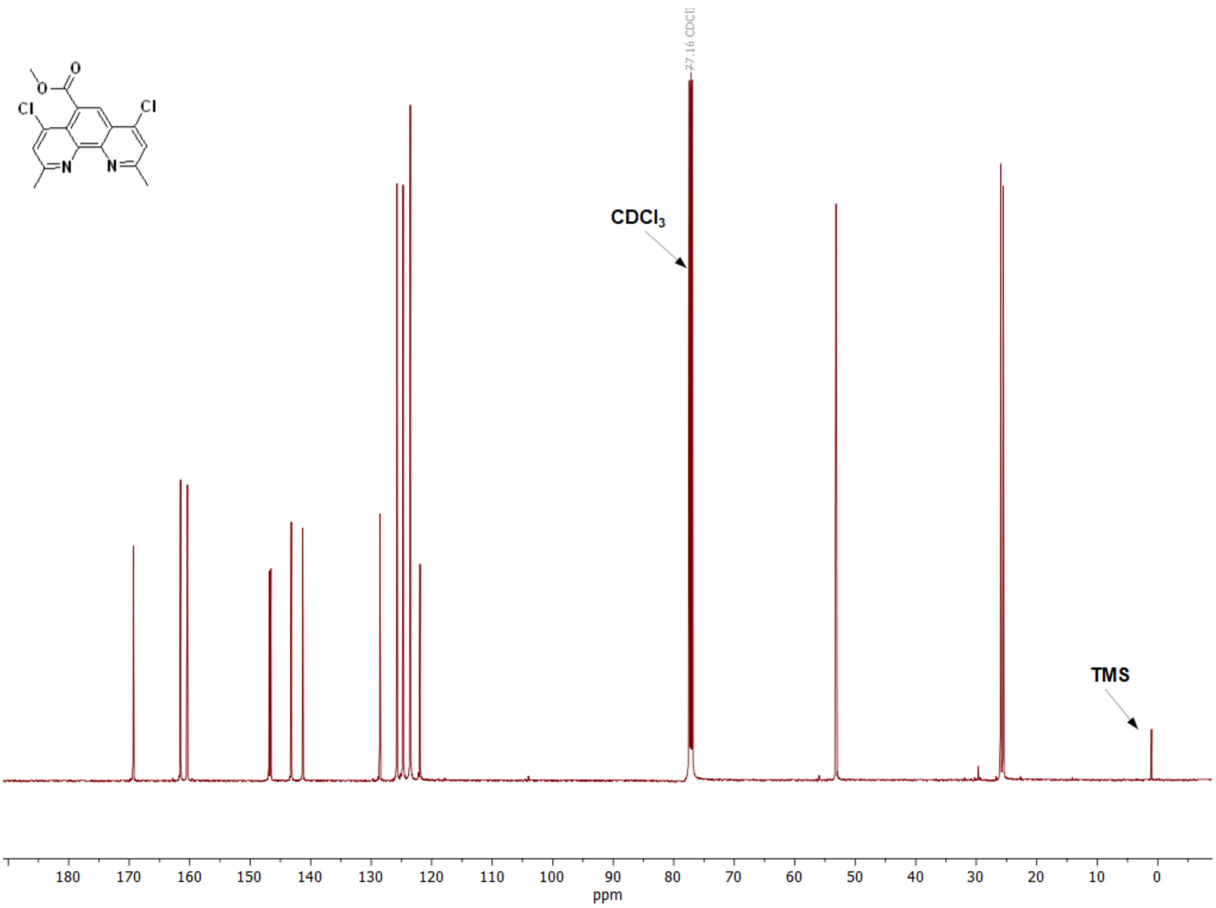

Fig. S3b. $^{13}C\{^{1}H\}$-NMR ($CDCl_3$; 125.8 MHz) spectrum of **1d**.

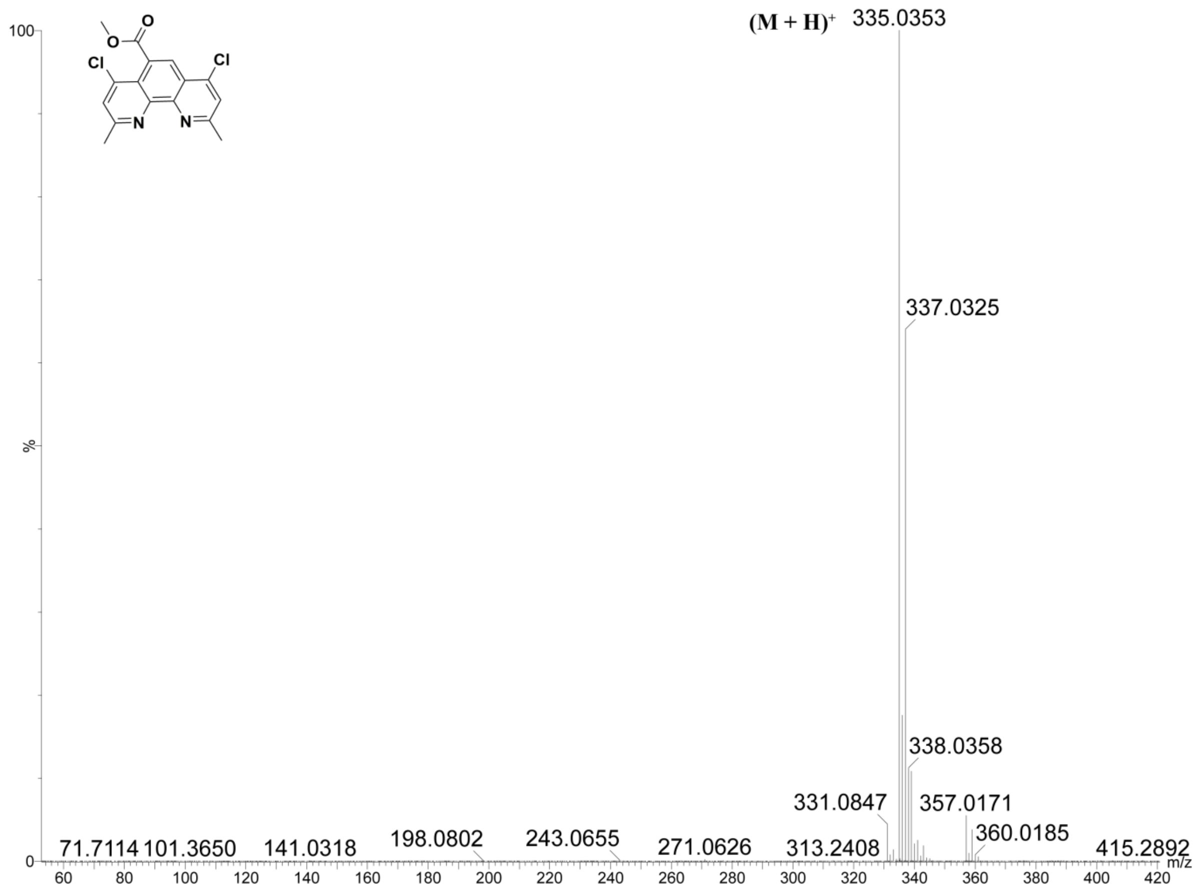

Fig. S3c. MS spectrum of **1d**.

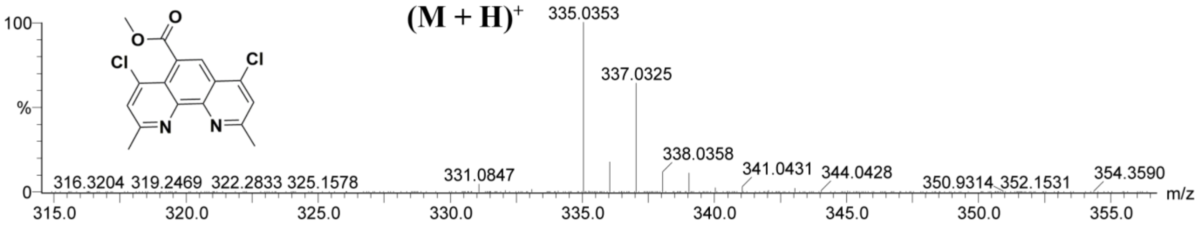

Fig. S3d. HRMS spectrum of **1d**.

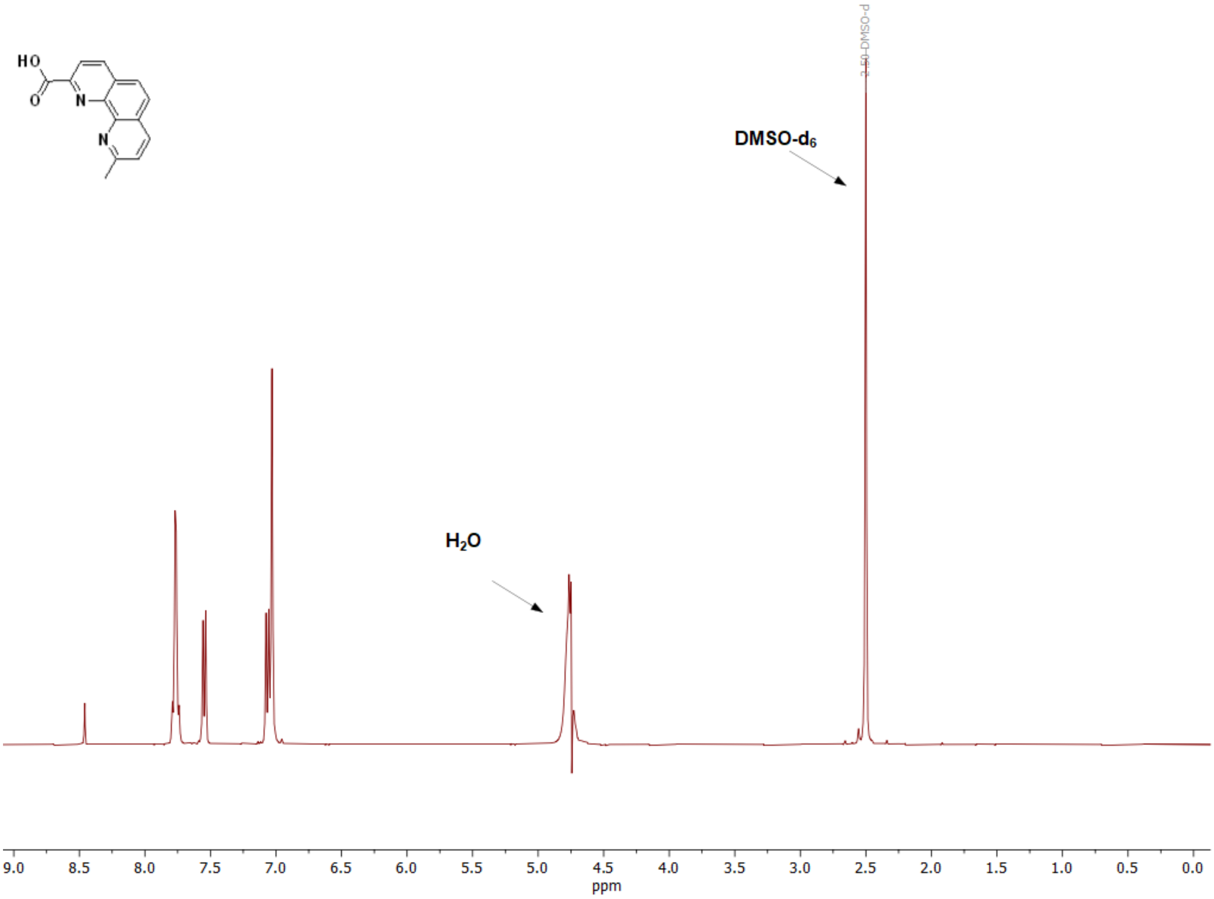

Fig. S4a. ^{1}H-NMR (D_2O/KOD; 400.2 MHz) spectrum of **2a**.

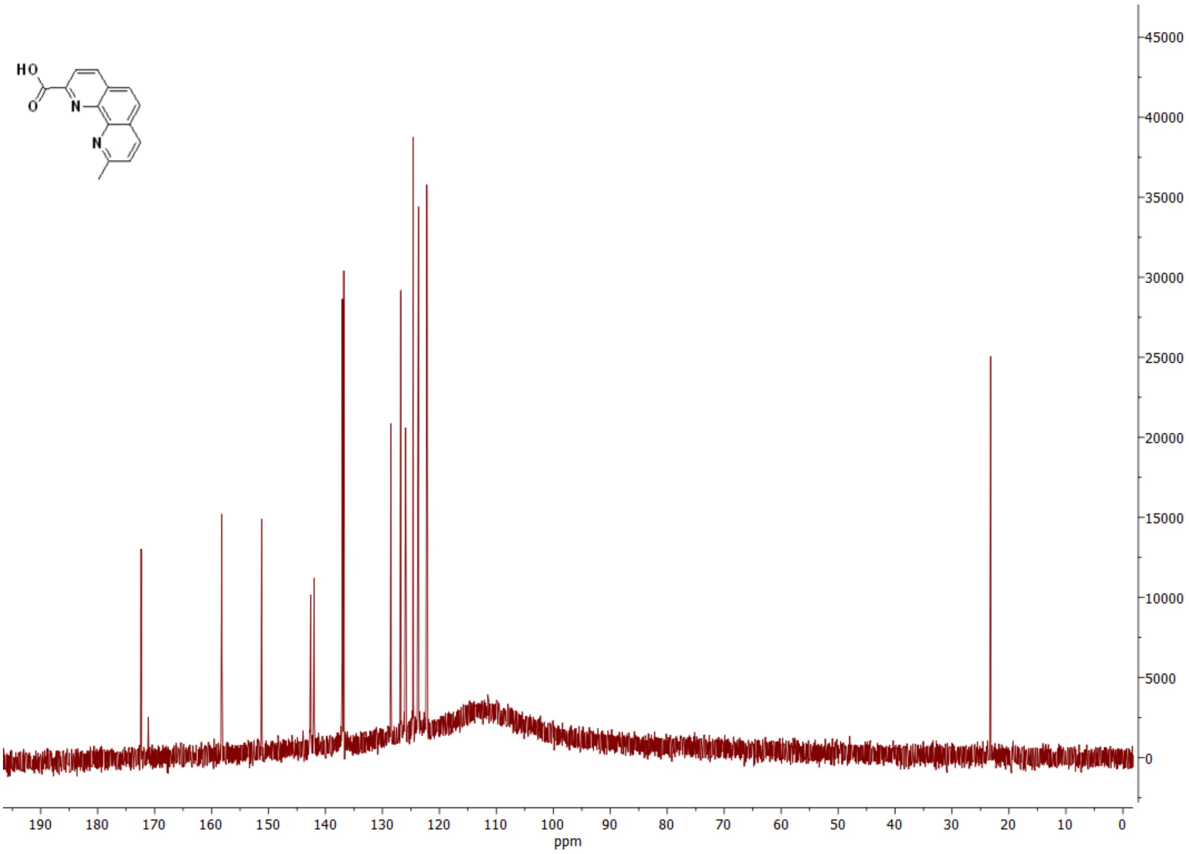

Fig. S4b. $^{13}C\{^1H\}$-NMR (D_2O; 100.5 MHz) spectrum of **2a**.

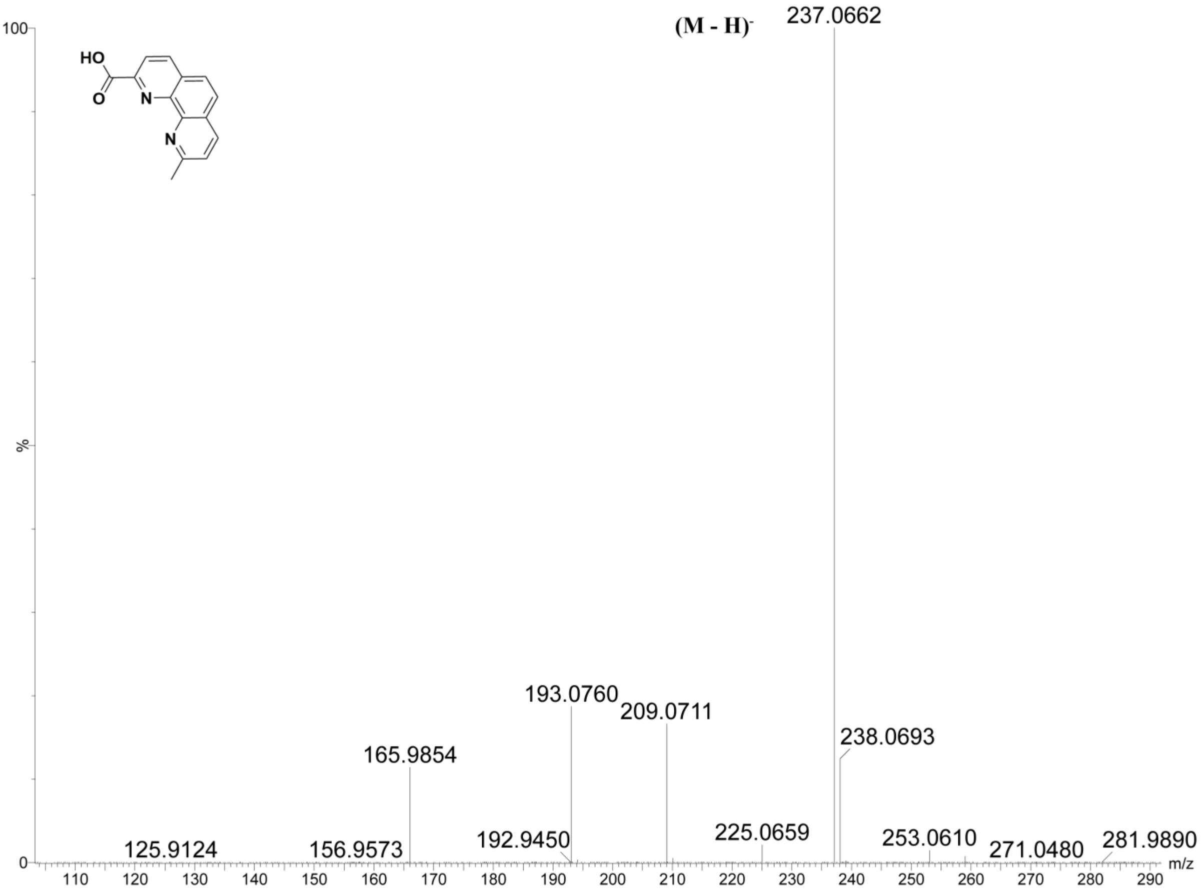

Fig. S4c. MS spectrum of **2a**.

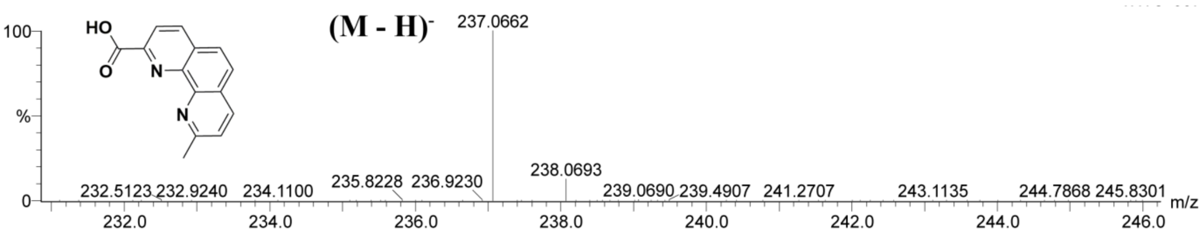

Fig. S4d. HRMS spectrum of **2a**.

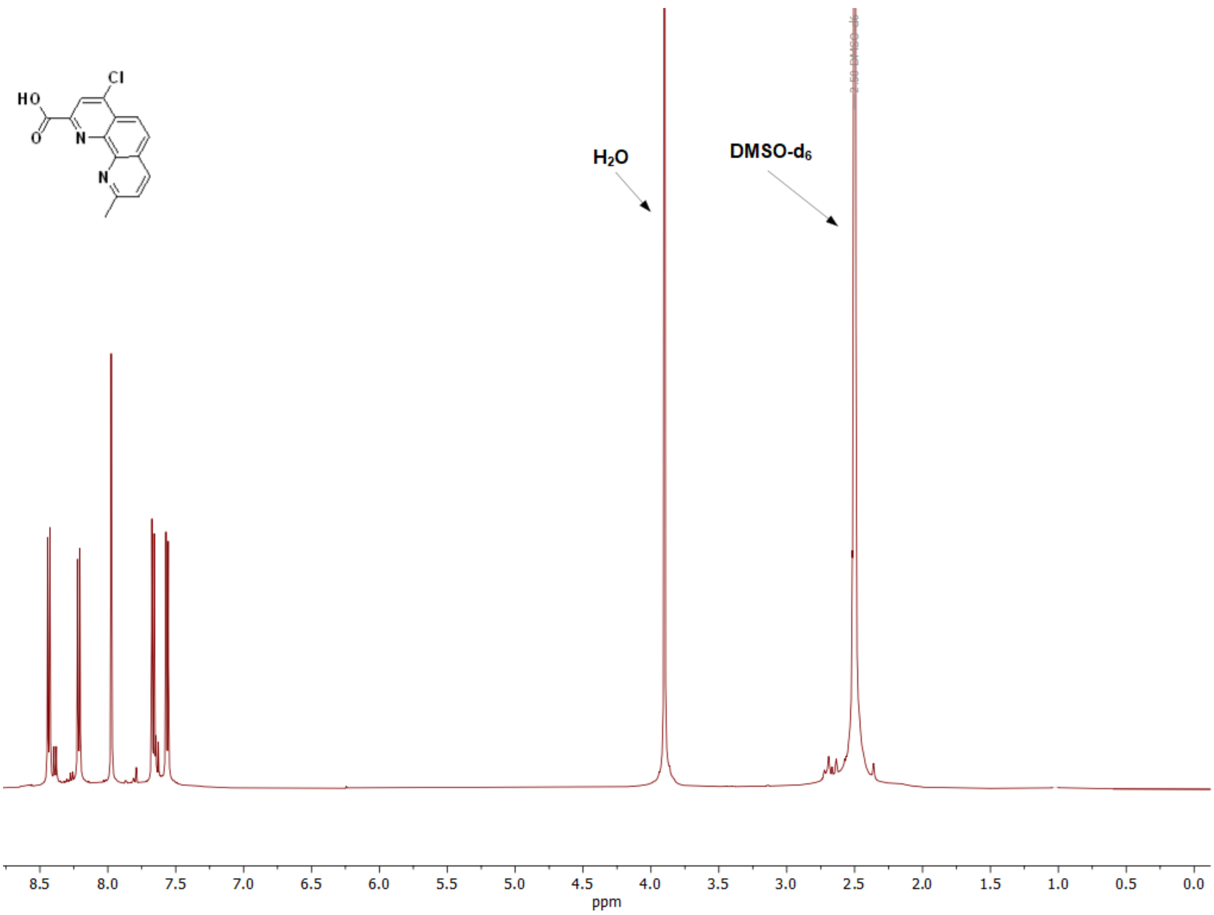

Fig. S5a. ^{1}H-NMR (DMSO-d$_6$/D$_2$O/KOD; 500.2 MHz) spectrum of **2b**.

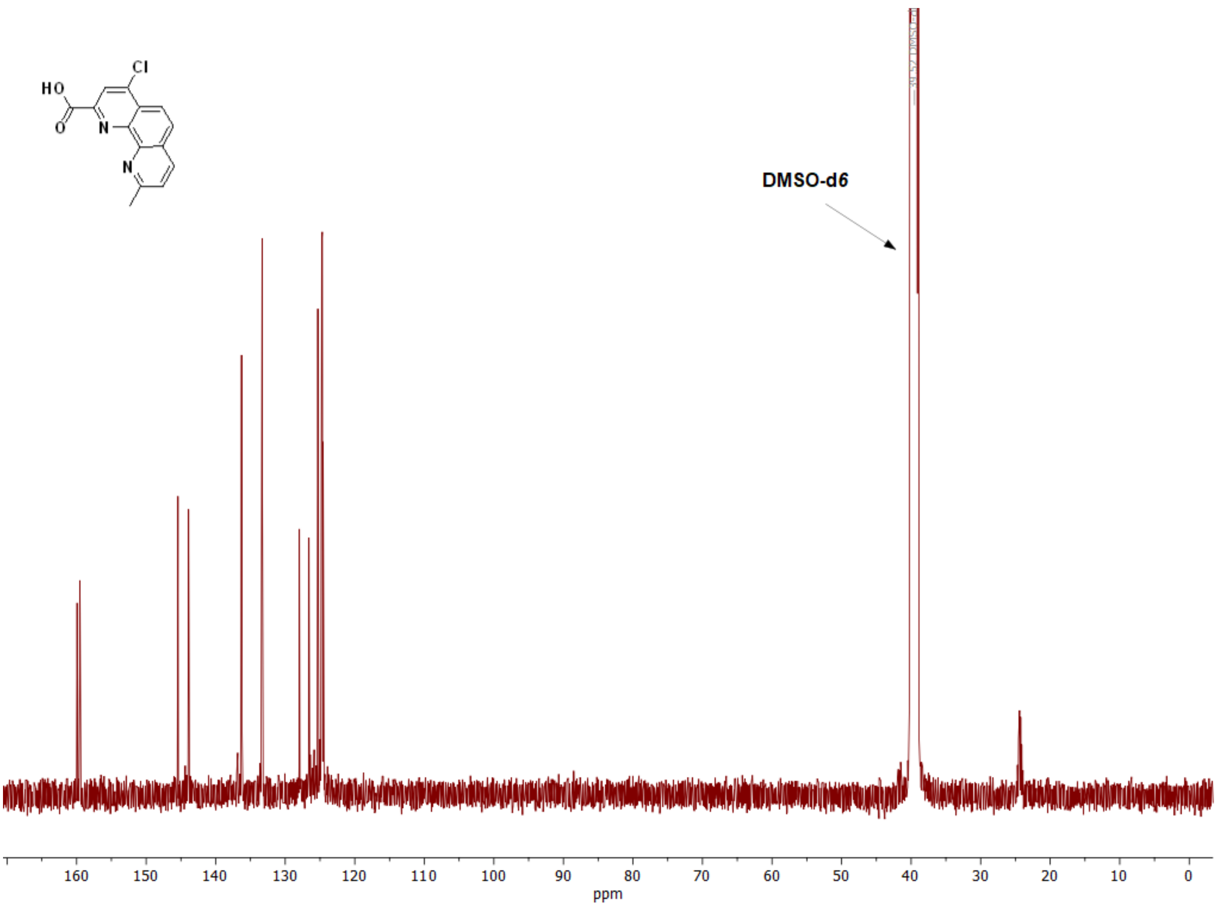

Fig. S5b. $^{13}C\{^{1}H\}$-NMR (DMSO-d_6/D_2O/KOD; 125.8 MHz) spectrum of **2b**.

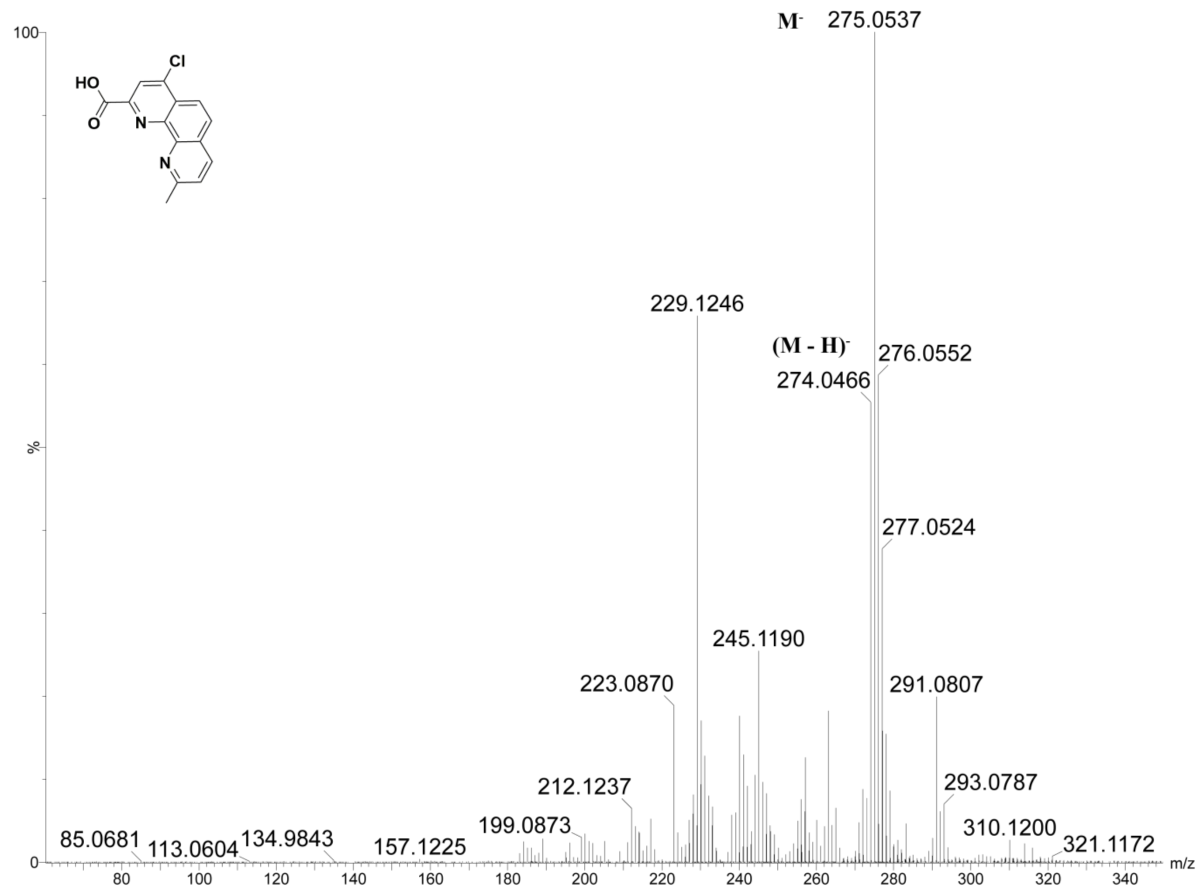

Fig. S5c. MS spectrum of **2b**.

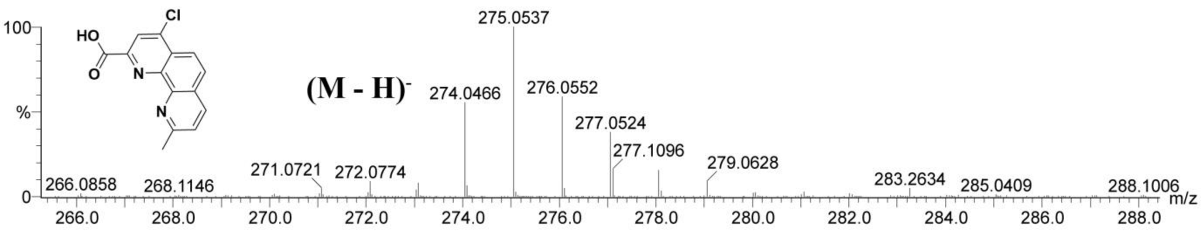

Fig. S5d. HRMS spectrum of **2b**.

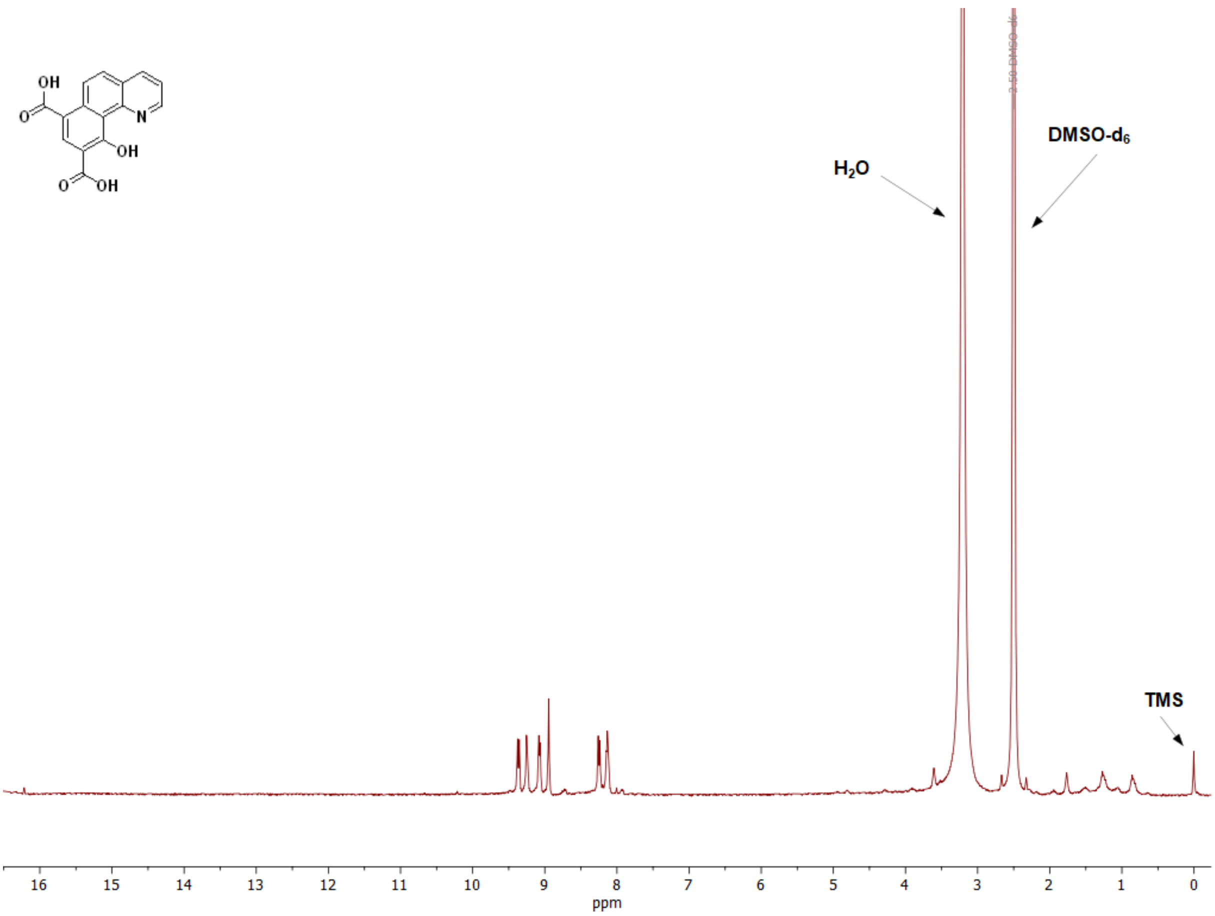

Fig. S6a. ^{1}H-NMR (DMSO-d$_6$; 400.2 MHz, 60 °C) spectrum of **3a**.

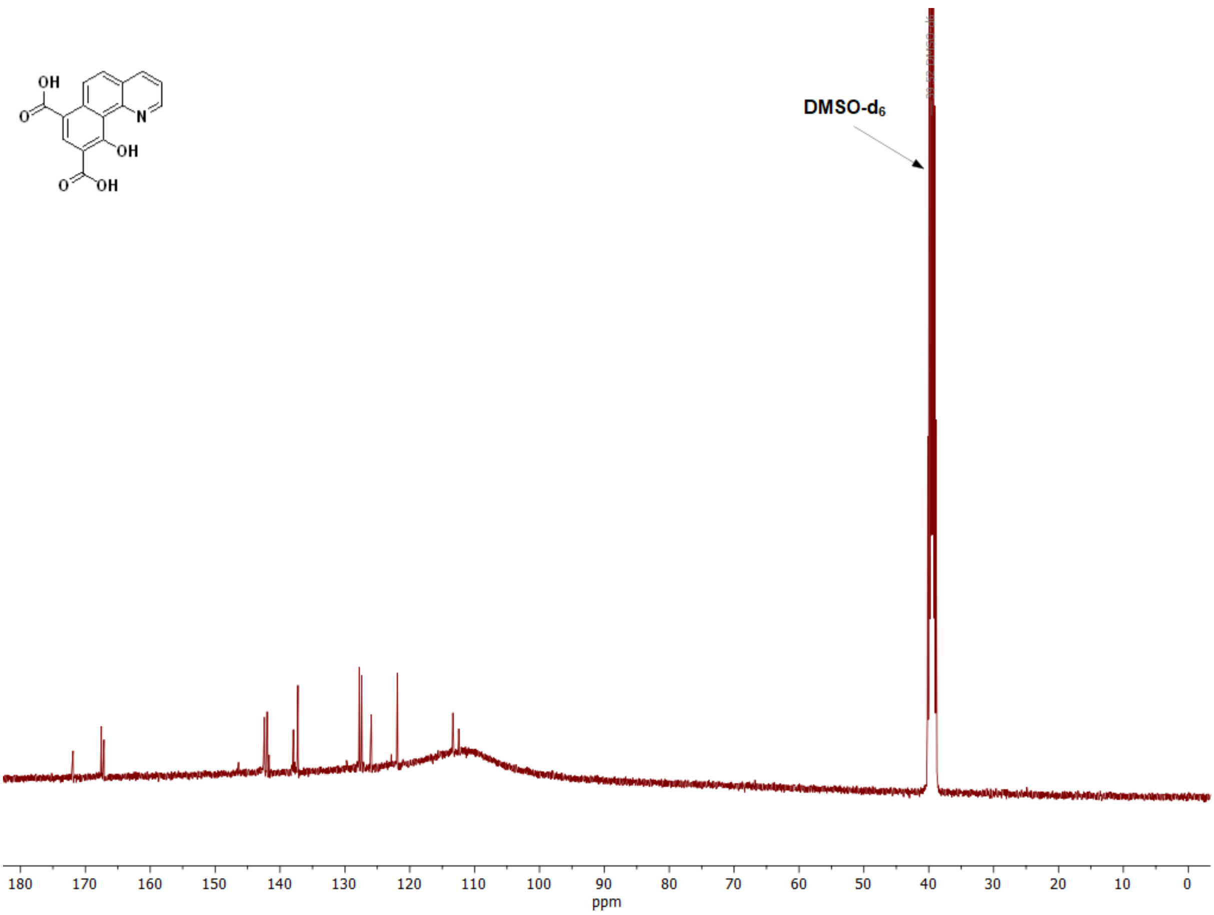

Fig. S6b. $^{13}C\{^1H\}$-NMR (DMSO-d_6; 100.6 MHz, 60 °C) spectrum of **3a**.

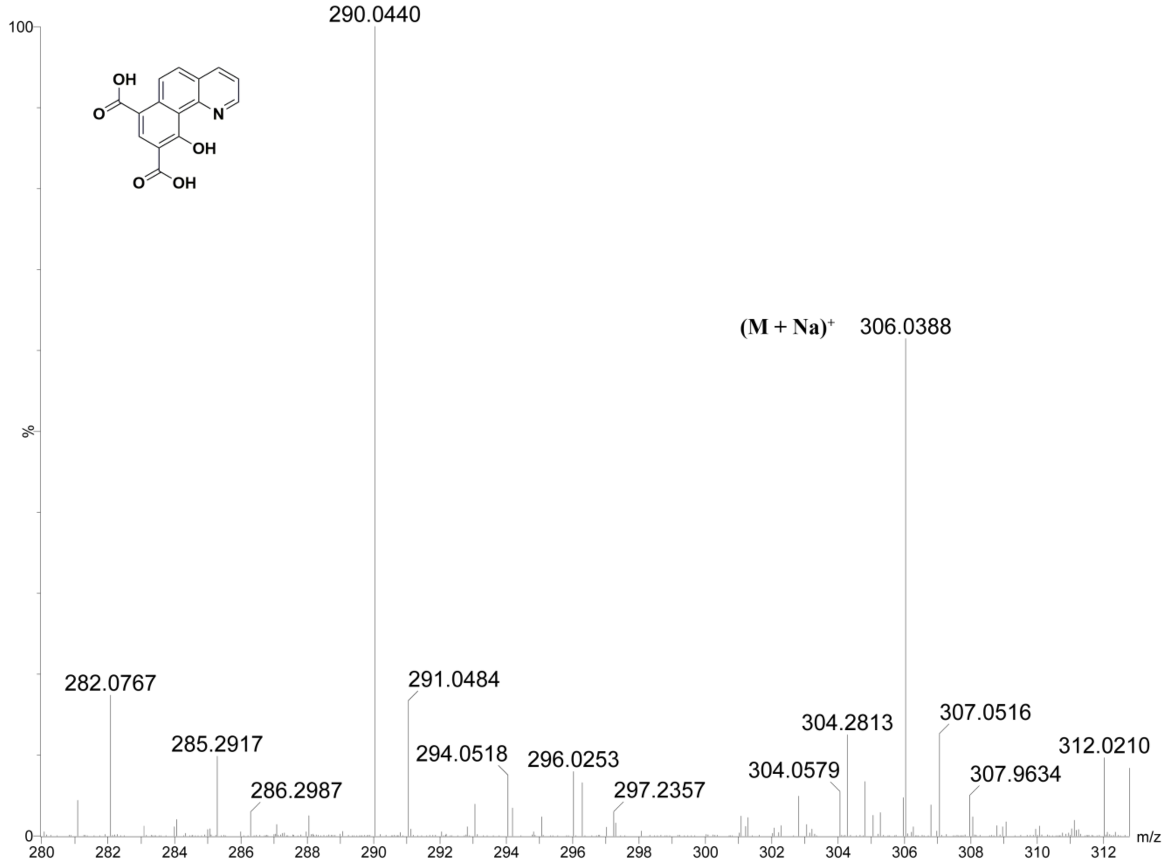

Fig. S6c. MS spectrum of **3a**.

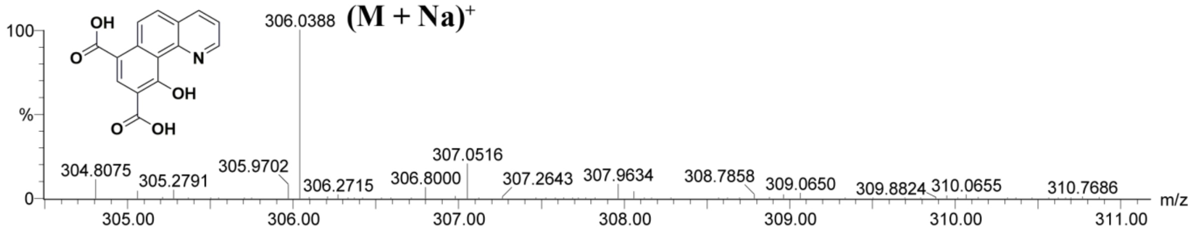

Fig. S6d. HRMS spectrum of **3a**.

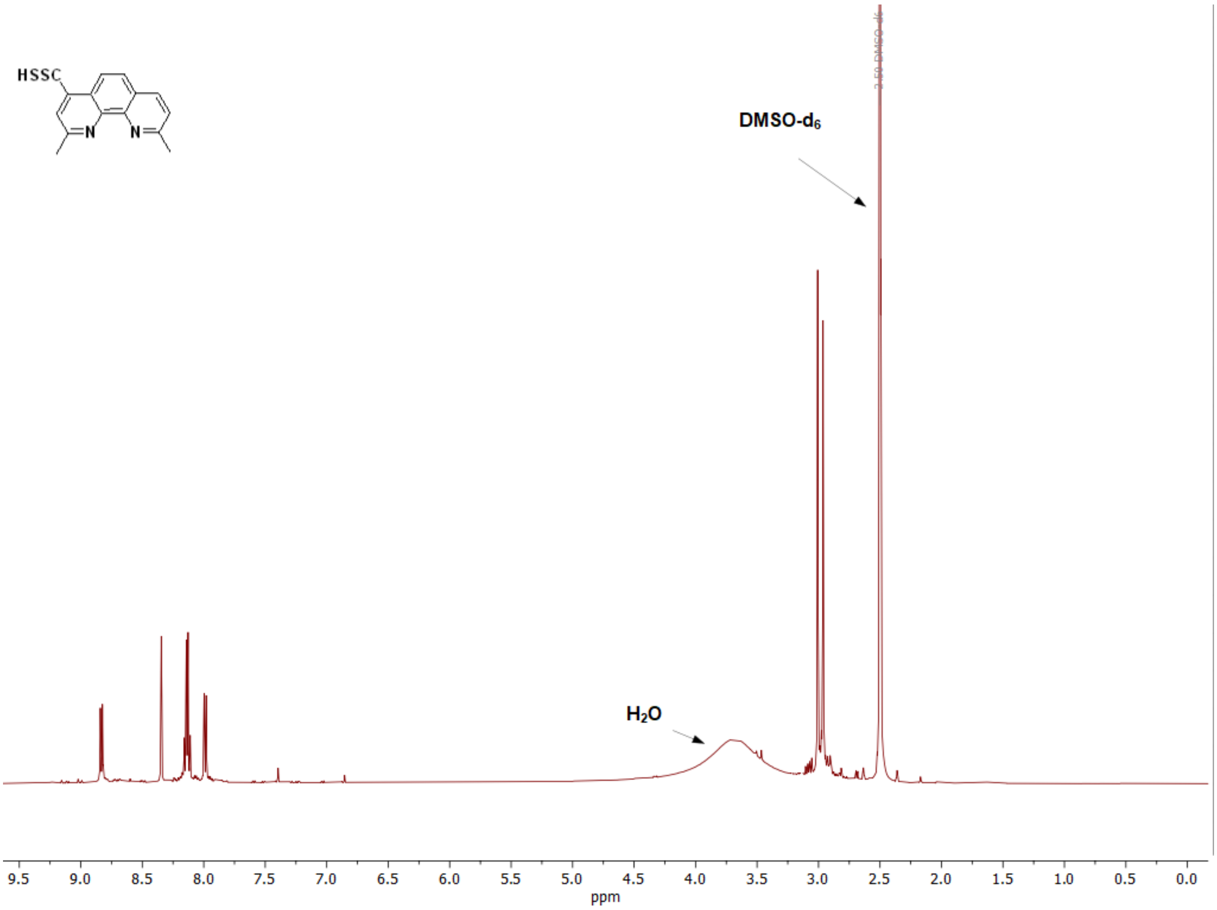

Fig. S7a. ^{1}H-NMR (DMSO-d6; 500.2 MHz) spectrum of **5a**.

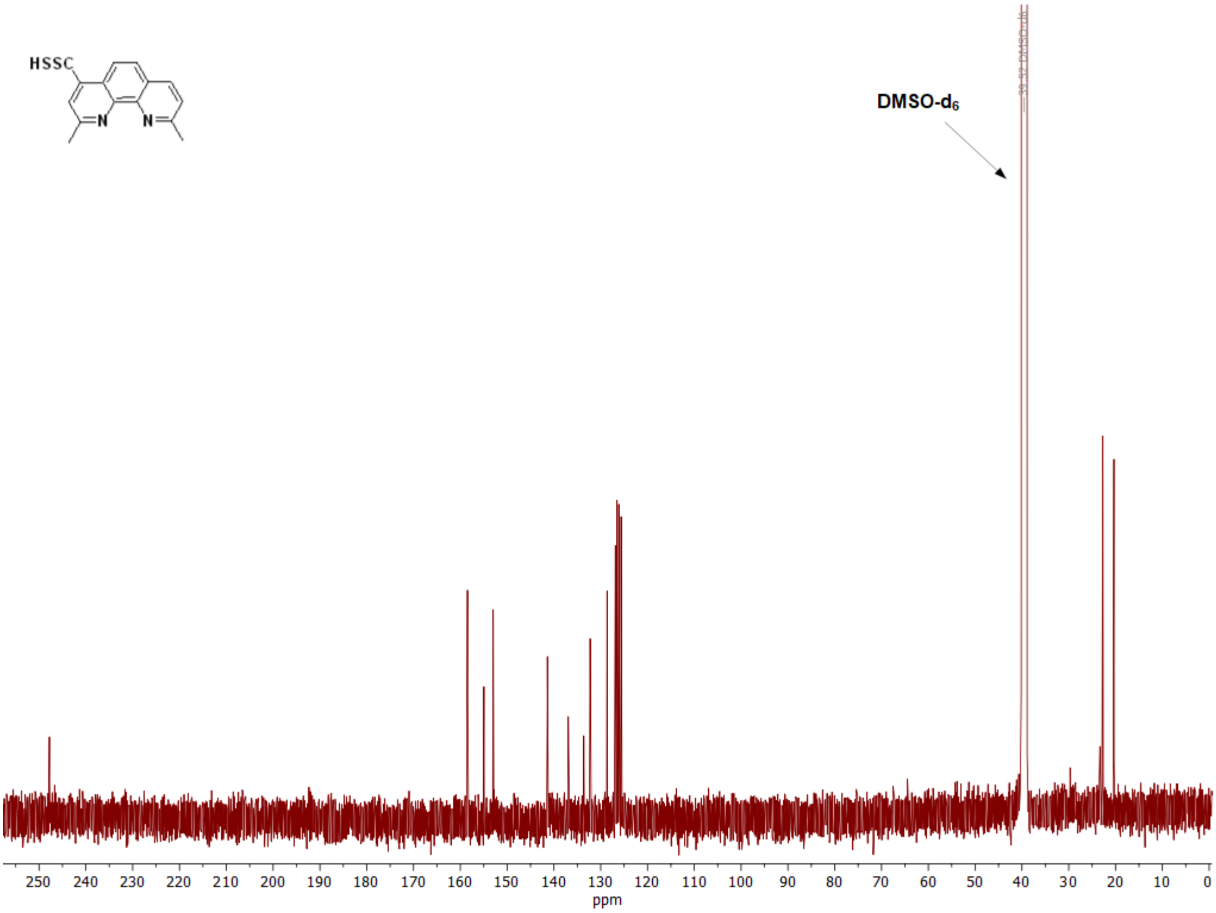

Fig. S7b. $^{13}C\{^1H\}$-NMR (DMSO-d$_6$; 125.8 MHz) spectrum of **5a**.

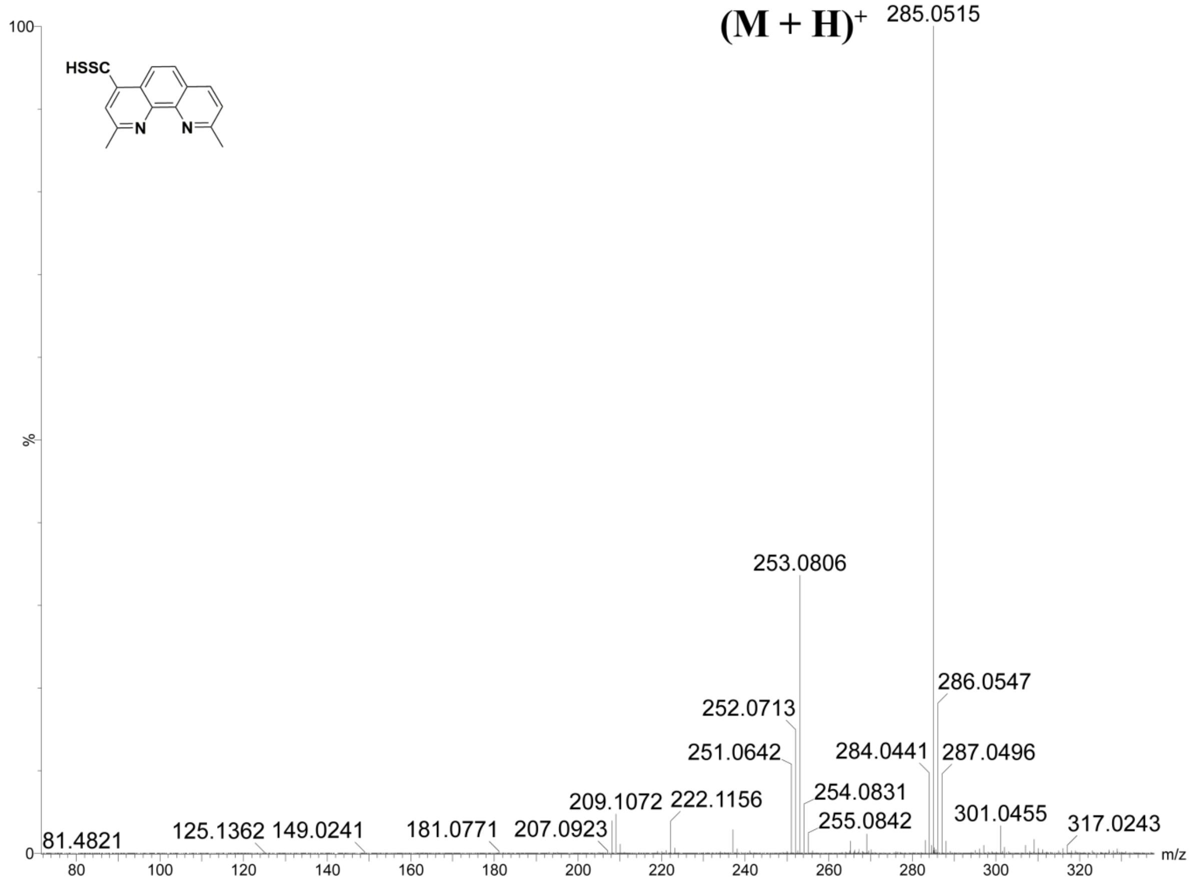

Fig. S7c. MS spectrum of **5a**.

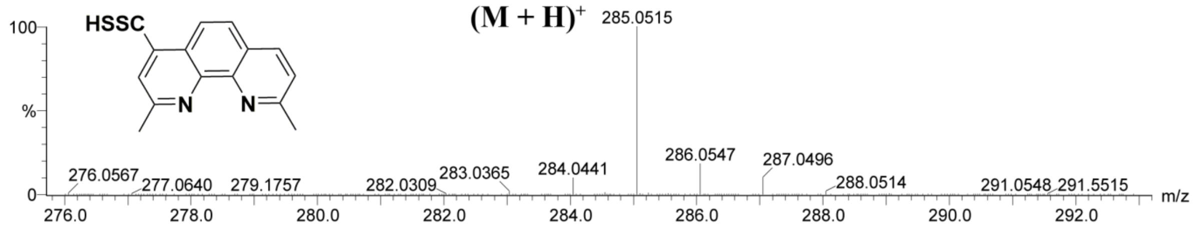

Fig. S7d. HRMS spectrum of **5a**.

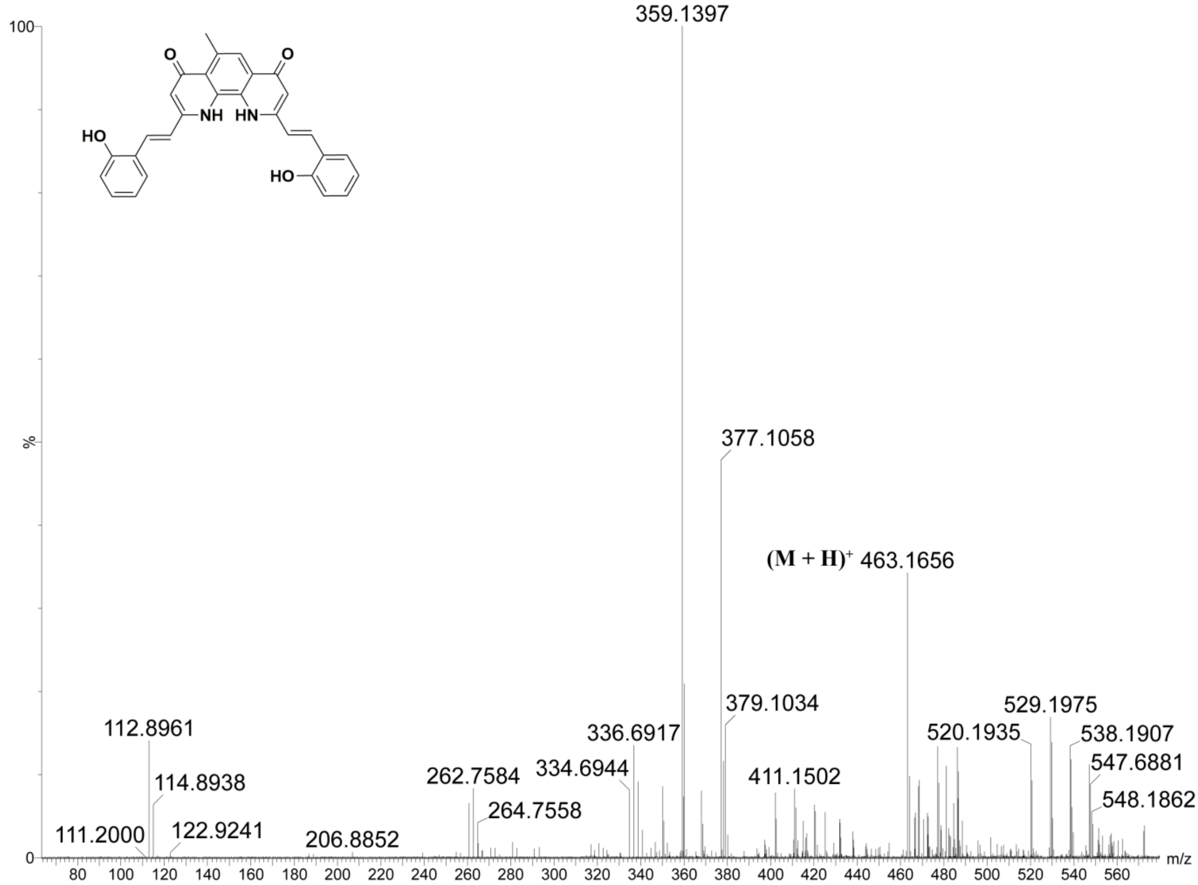

Fig. S8a. MS spectrum of **6a**.

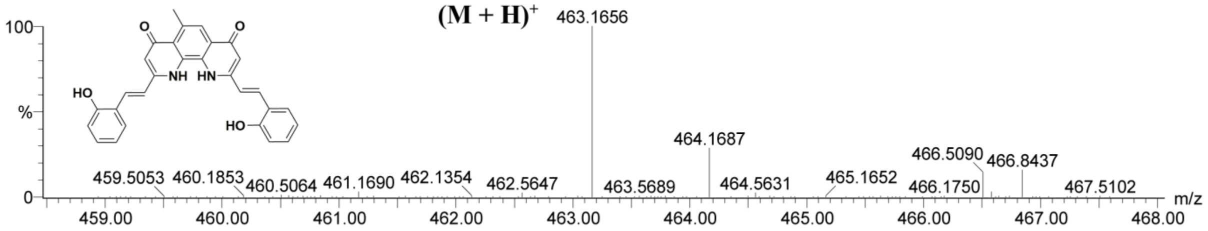

Fig. S8b. HRMS spectrum of **6a**.

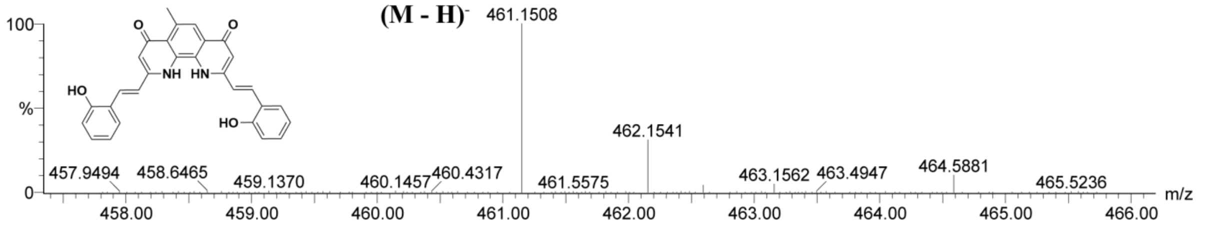

Fig. S8c. HRMS spectrum of **6a**.

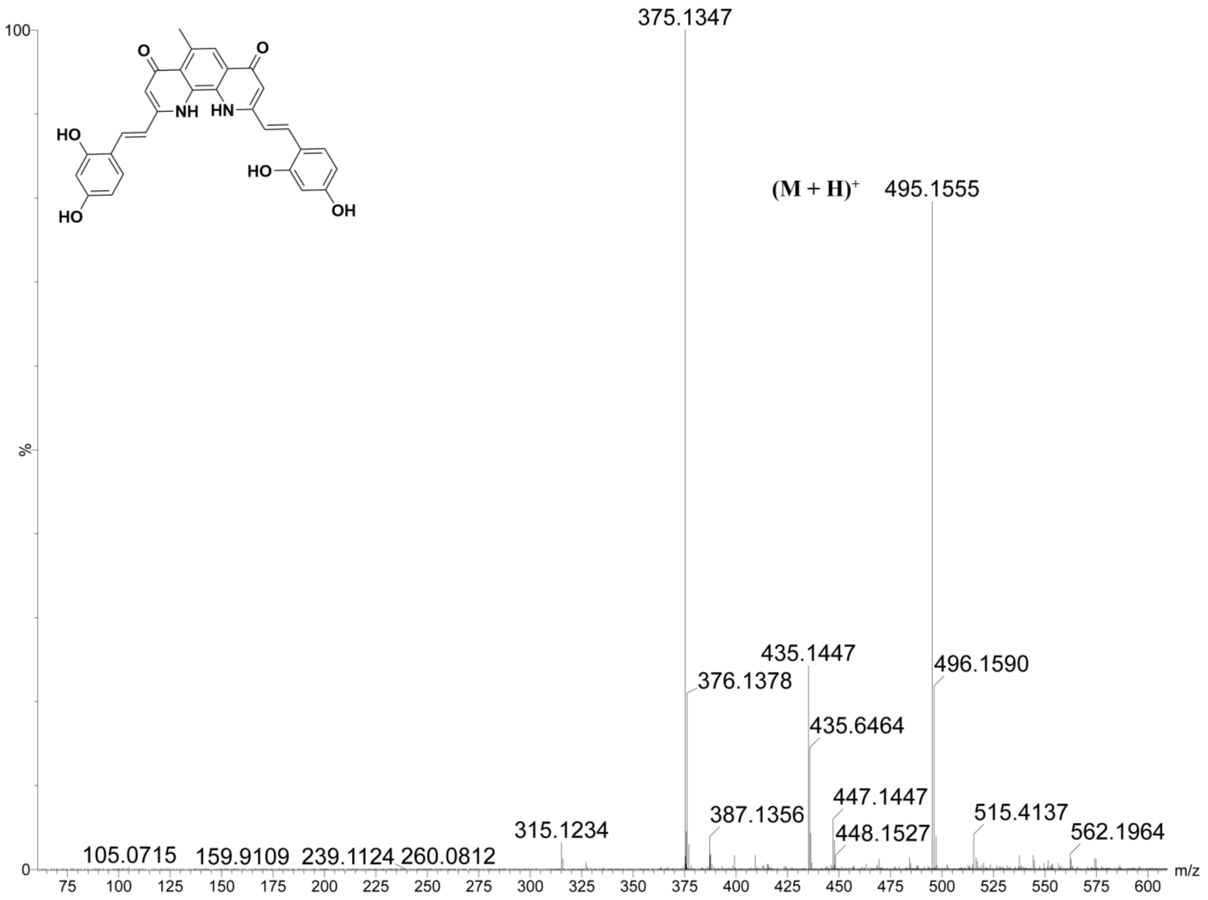

Fig. S9a. MS spectrum of **6b**.

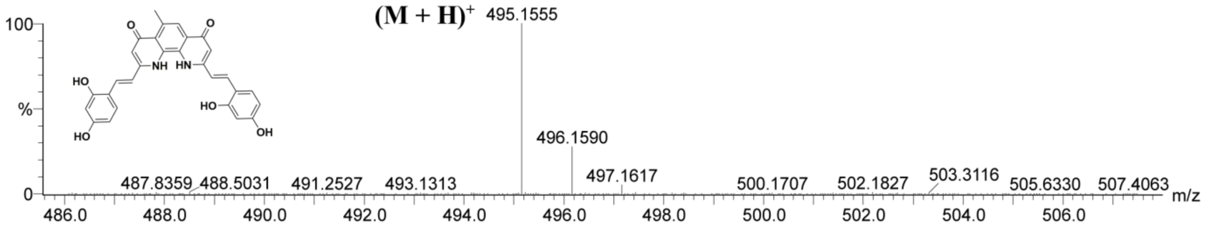

Fig. S9b. HRMS spectrum of **6b**.

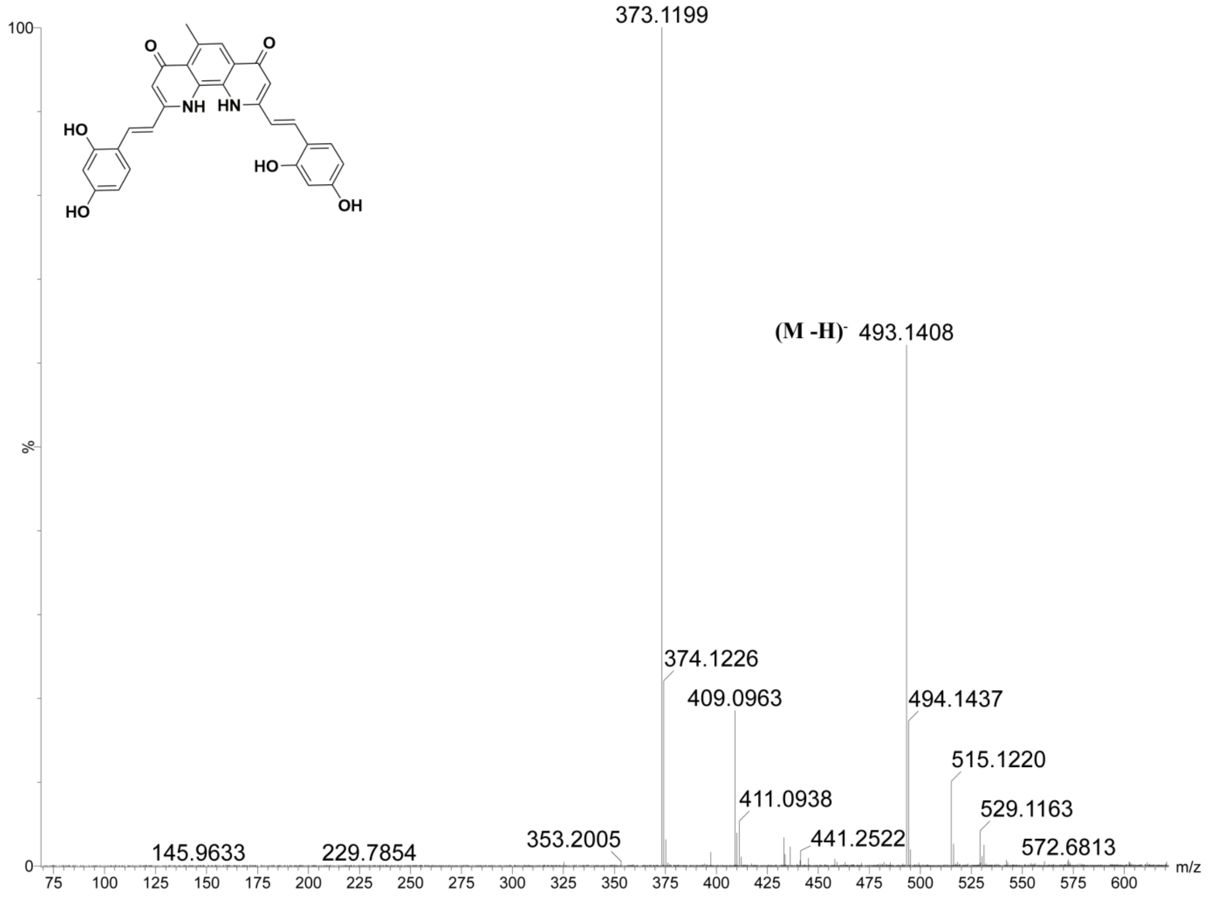

Fig. S9c. MS spectrum of **6b**.

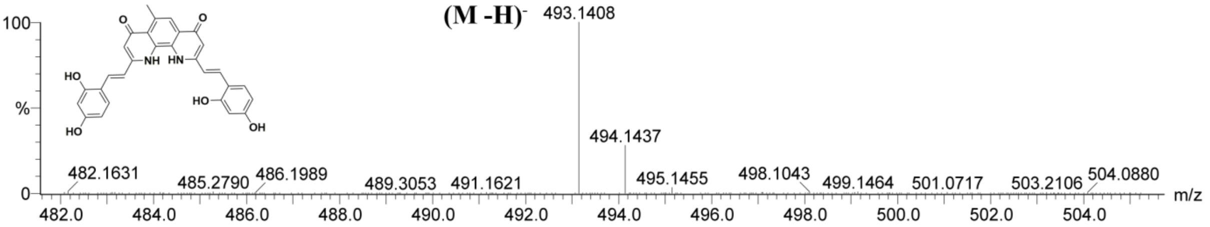

Fig. S9d. HRMS spectrum of **6b**.

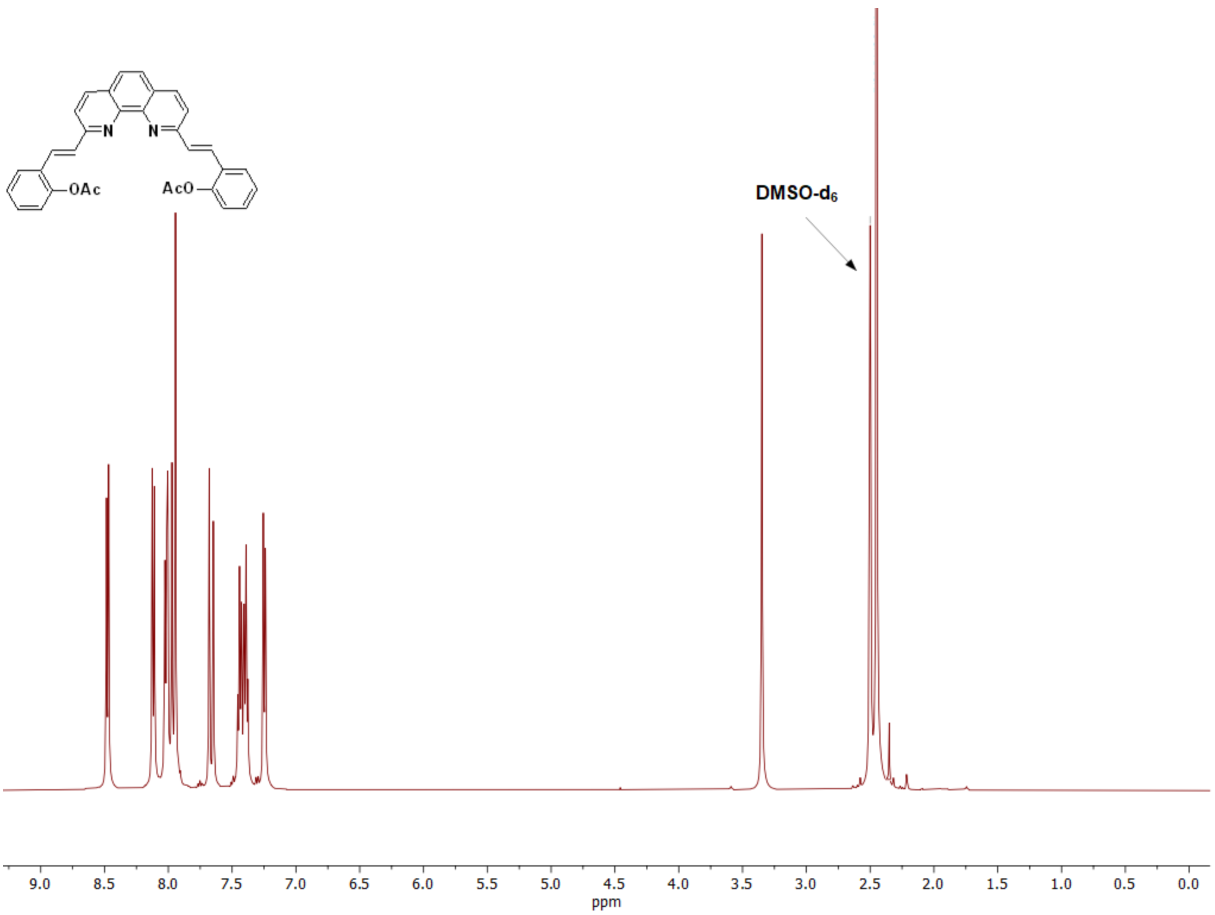

Fig. S10a. ^{1}H-NMR (DMSO-d6; 500.2 MHz) spectrum of **6c**.

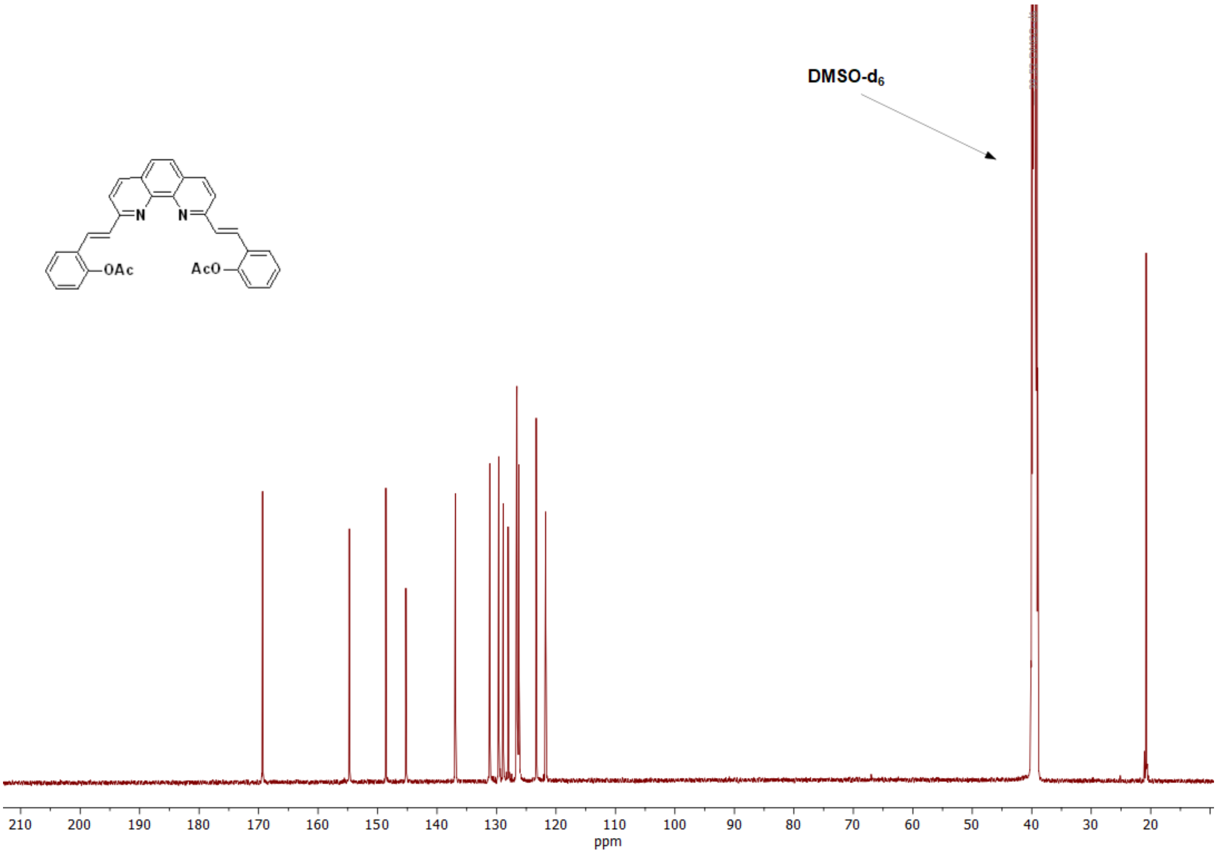

Fig. S10b. $^{13}C\{^{1}H\}$-NMR (DMSO-d$_6$; 125.8 MHz) spectrum of **6c**.

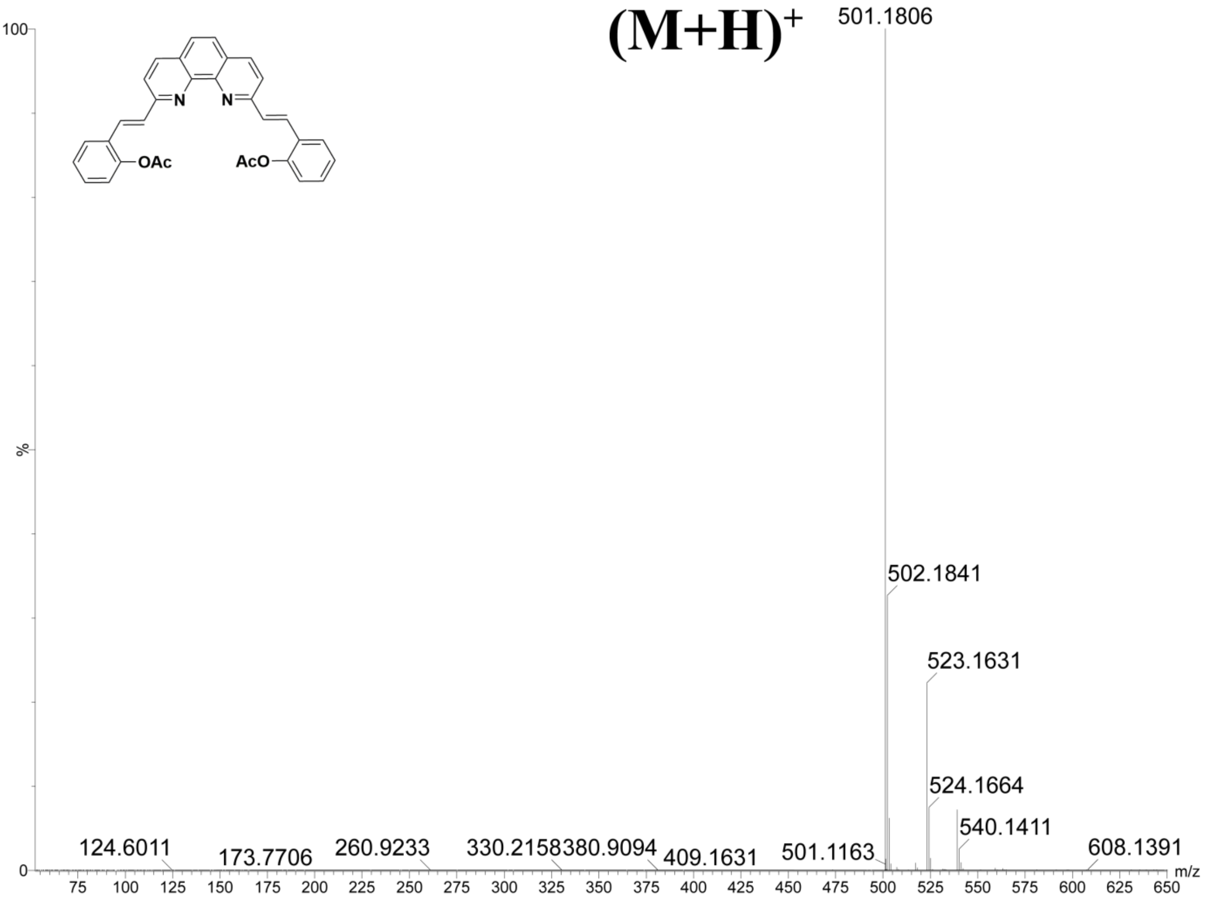

Fig. S10c. MS spectrum of **6c**.

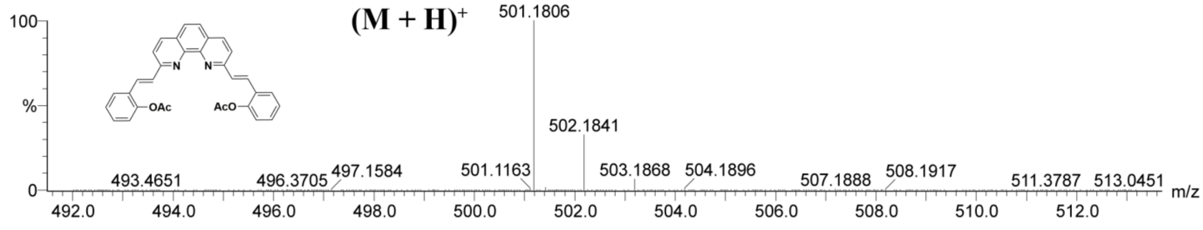

Fig. S10d. HRMS spectrum of **6c**.

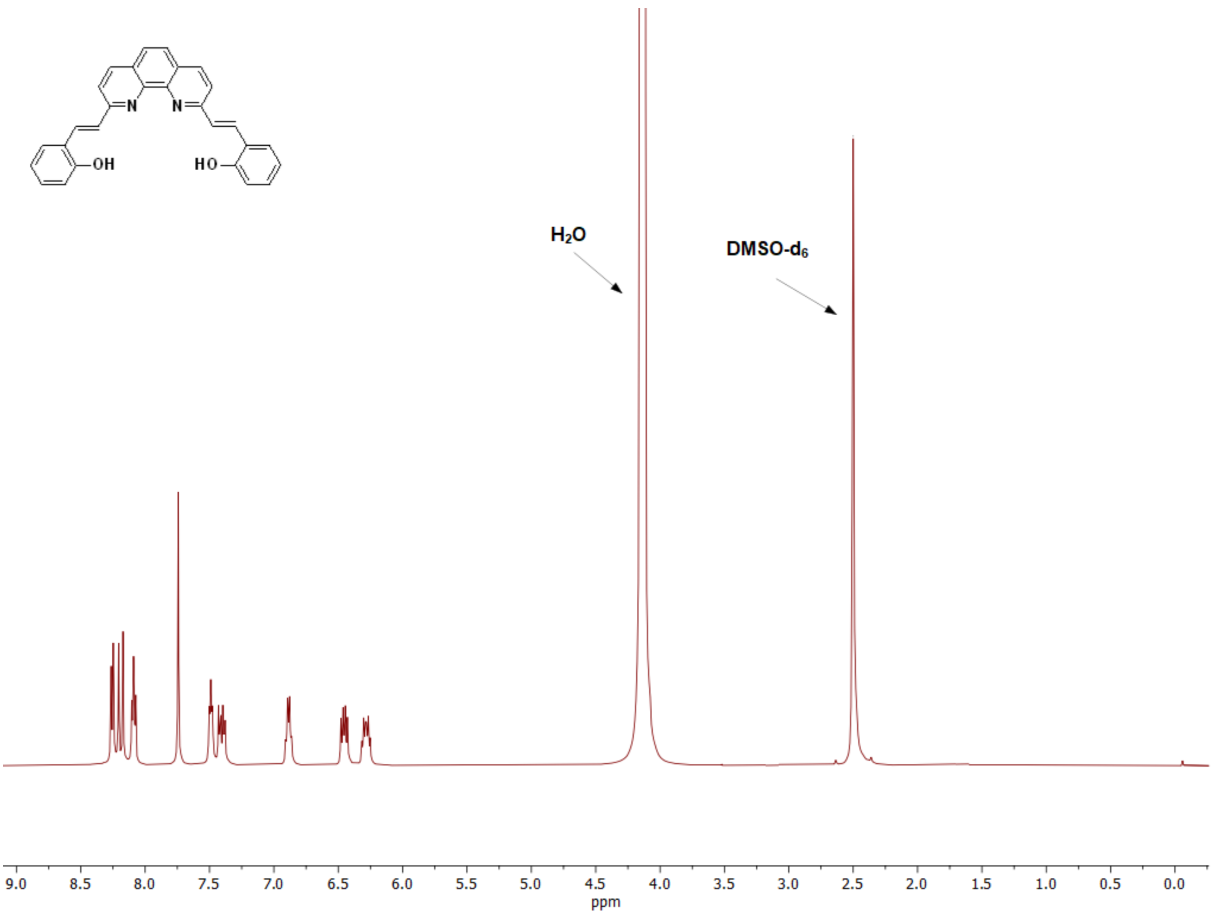

Fig. S11a. ^{1}H-NMR (DMSO-d$_6$/KOD; 500.2 MHz) spectrum of **6d**.

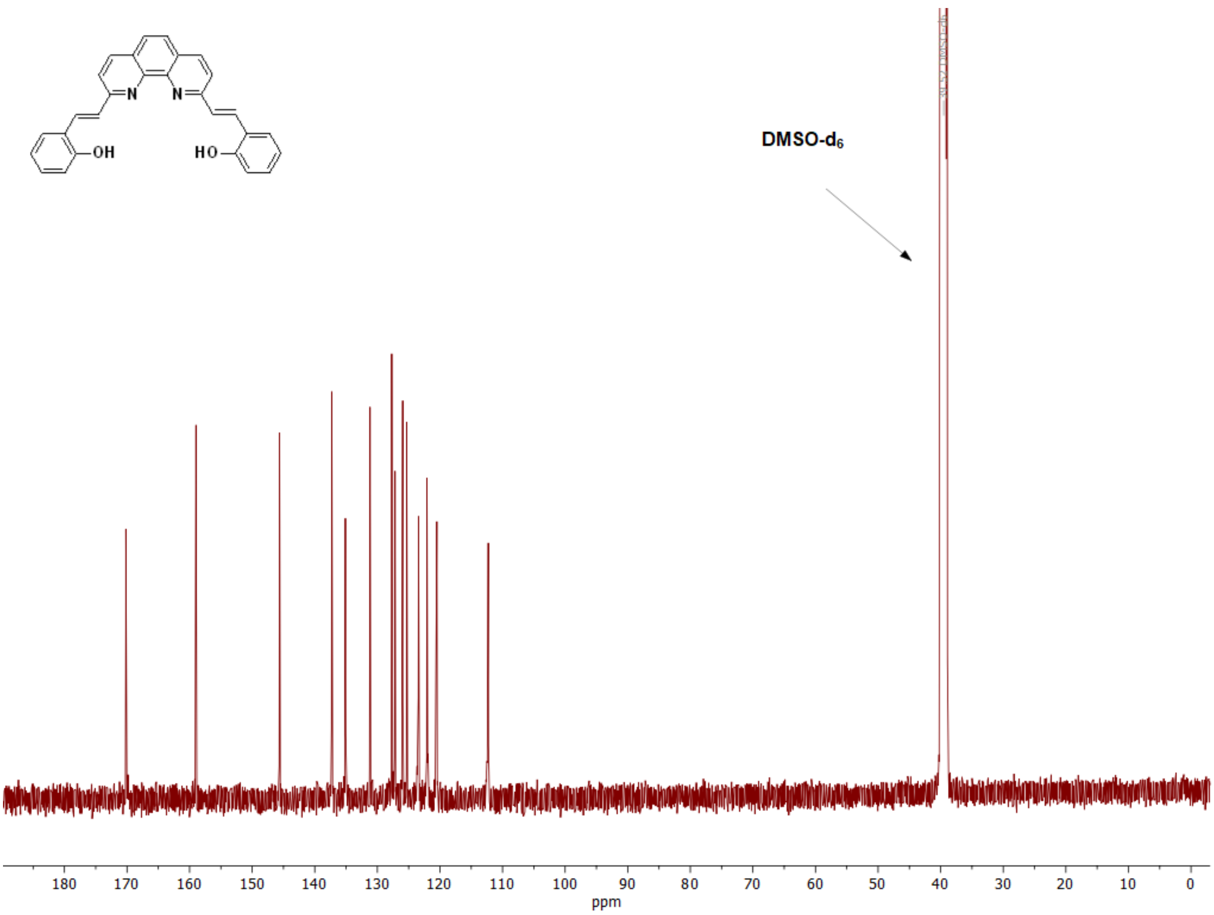

Fig. S11b. $^{13}C\{^{1}H\}$-NMR (DMSO-d_6/KOD; 125.8 MHz) spectrum of **6d**.

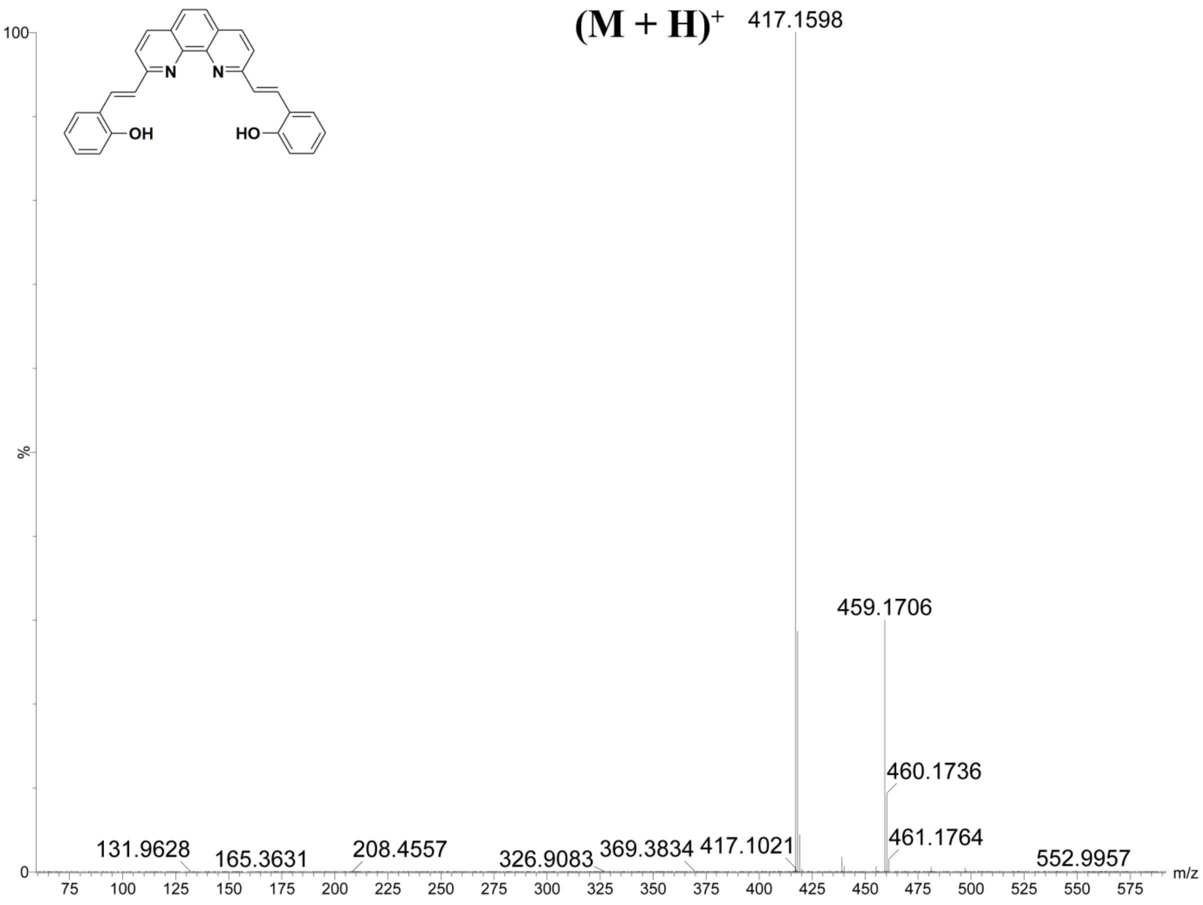

Fig. S11c. MS spectrum of **6d**.

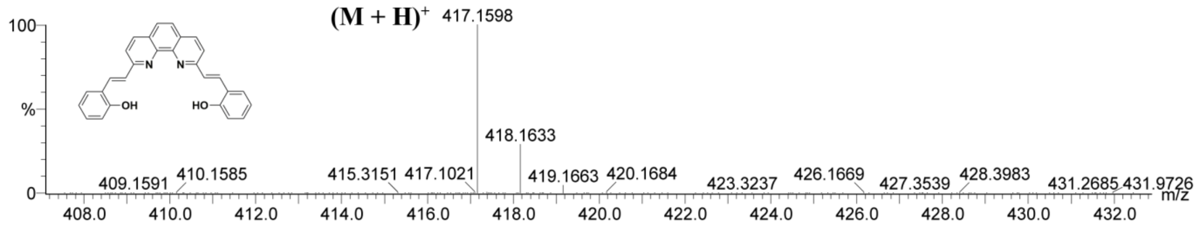

Fig. S11d. HRMS spectrum of **6d**.
